# Supplementary material for: Nanoscale triboelectrification gated transistor
Source: Nat Commun. 2020 Feb 26;11:1054. doi: 10.1038/s41467-020-14909-6 (PMC7044230; doi:10.1038/s41467-020-14909-6)
Supplement: Supplementary file 1 — Supplementary Information [file 41467_2020_14909_MOESM1_ESM.pdf]

Supplementary Information

Nanoscale Triboelectrification Gated Transistor

Bu et al.

Supplementary Figures

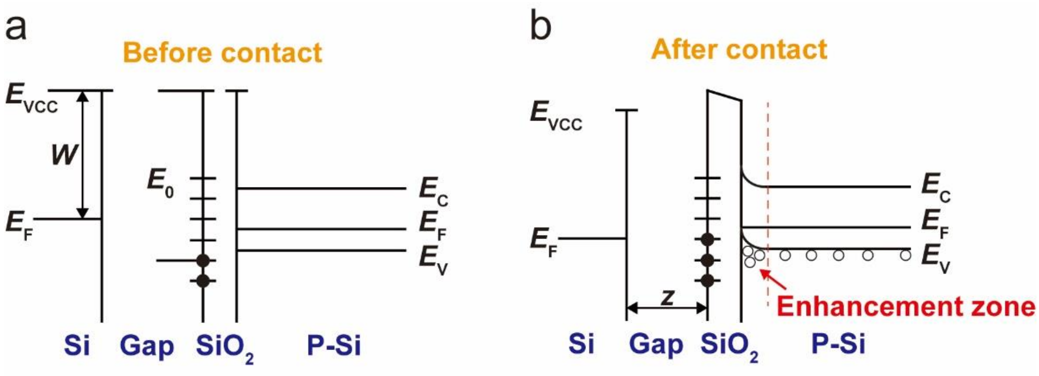

Supplementary Figure 1. Energy band diagrams of the nanoscale triboelectrification-gated transistor (NTT). a) The energy band diagram of the NTT before contact. b) The energy band diagram of the NTT after contact.

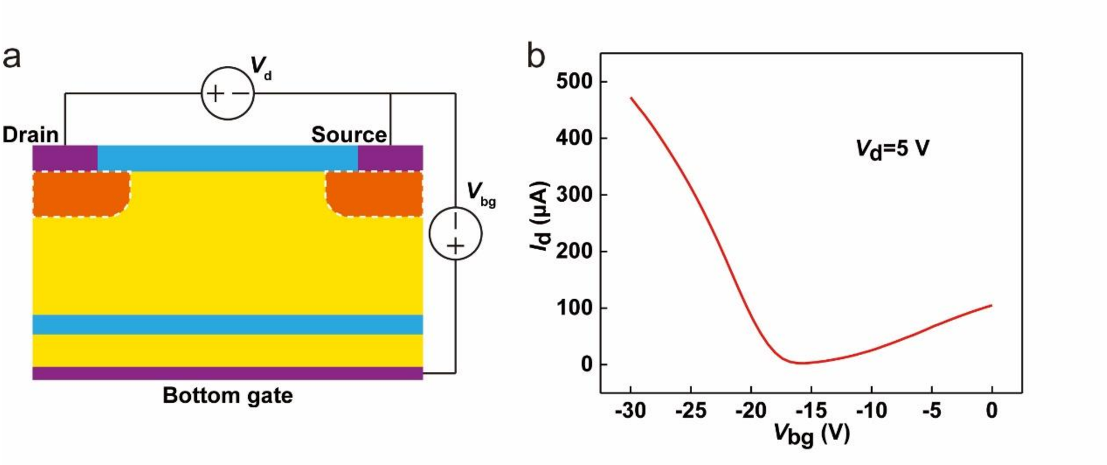

Supplementary Figure 2. Electrical characteristics of the transistor. a) Schematic circuit diagram of the transistor by applying an external bottom gate voltage. b) The corresponding I_d-V_{bg} transfer characteristics of the transistor at a drain voltage of 5 V.

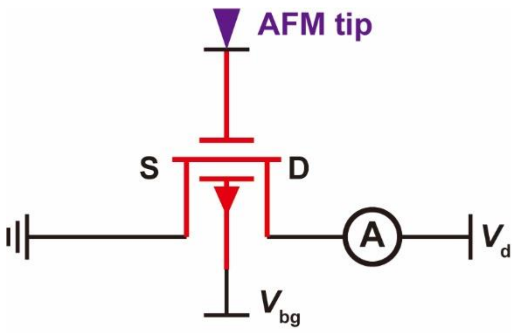

Supplementary Figure 3. The equivalent circuit of the NTT.

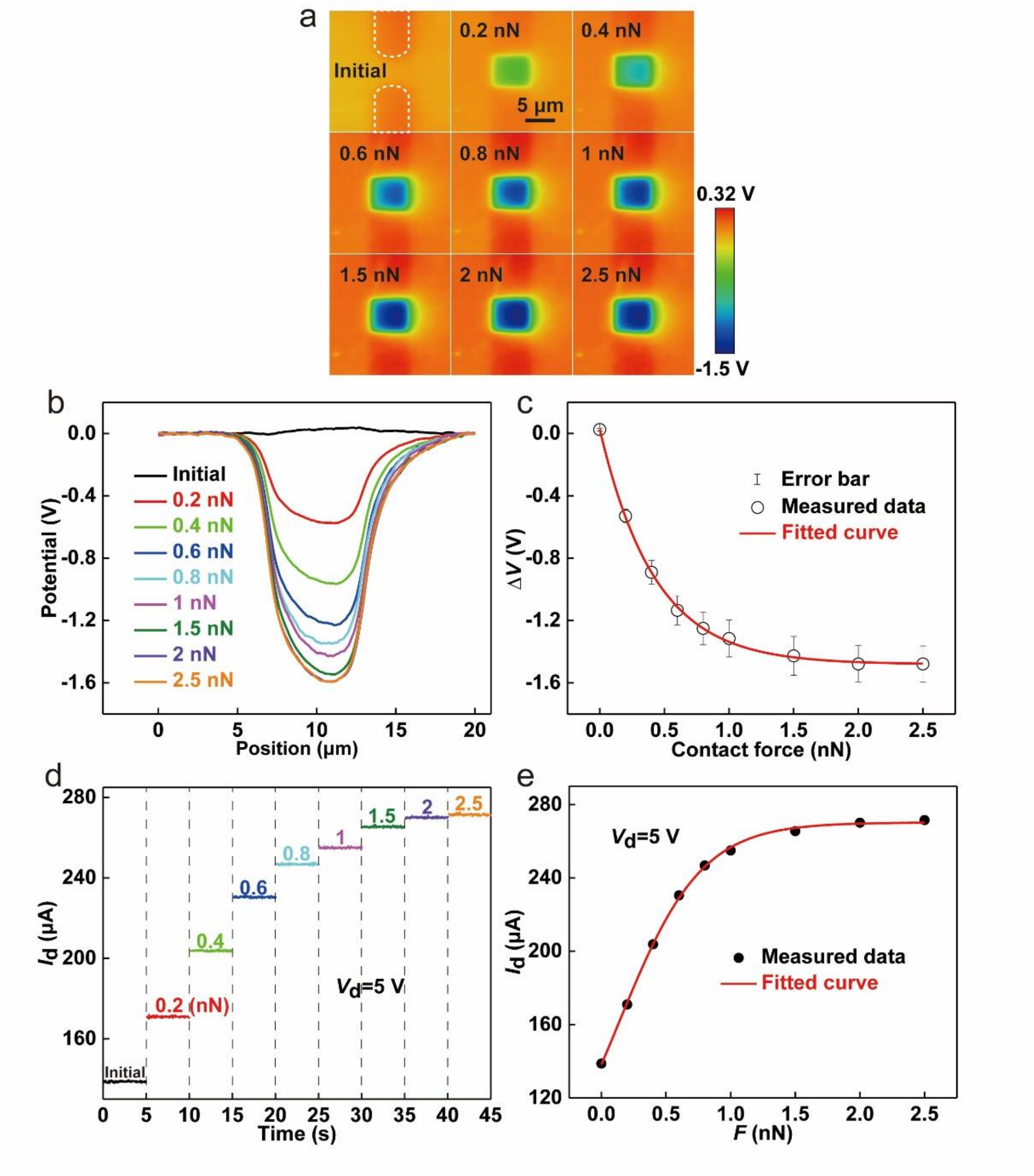

Supplementary Figure 4. The effect of contact force on the characteristics of the NTT. a) Surface potential distributions of the NTT after regionally rubbed by the atomic force microscopy (AFM) tip with increasing contact force. b) Corresponding potential distributions in cross-sectional view with different contact forces. c) The potential difference between the rubbed and surrounding area with different contact forces. d) I_d output characterisics at a drain voltage of 5 V with different contact forces from 0 to 2.5 nN. e) The I_d-F transfer characteristics. All error bars in the figure represent s.d. of the data.

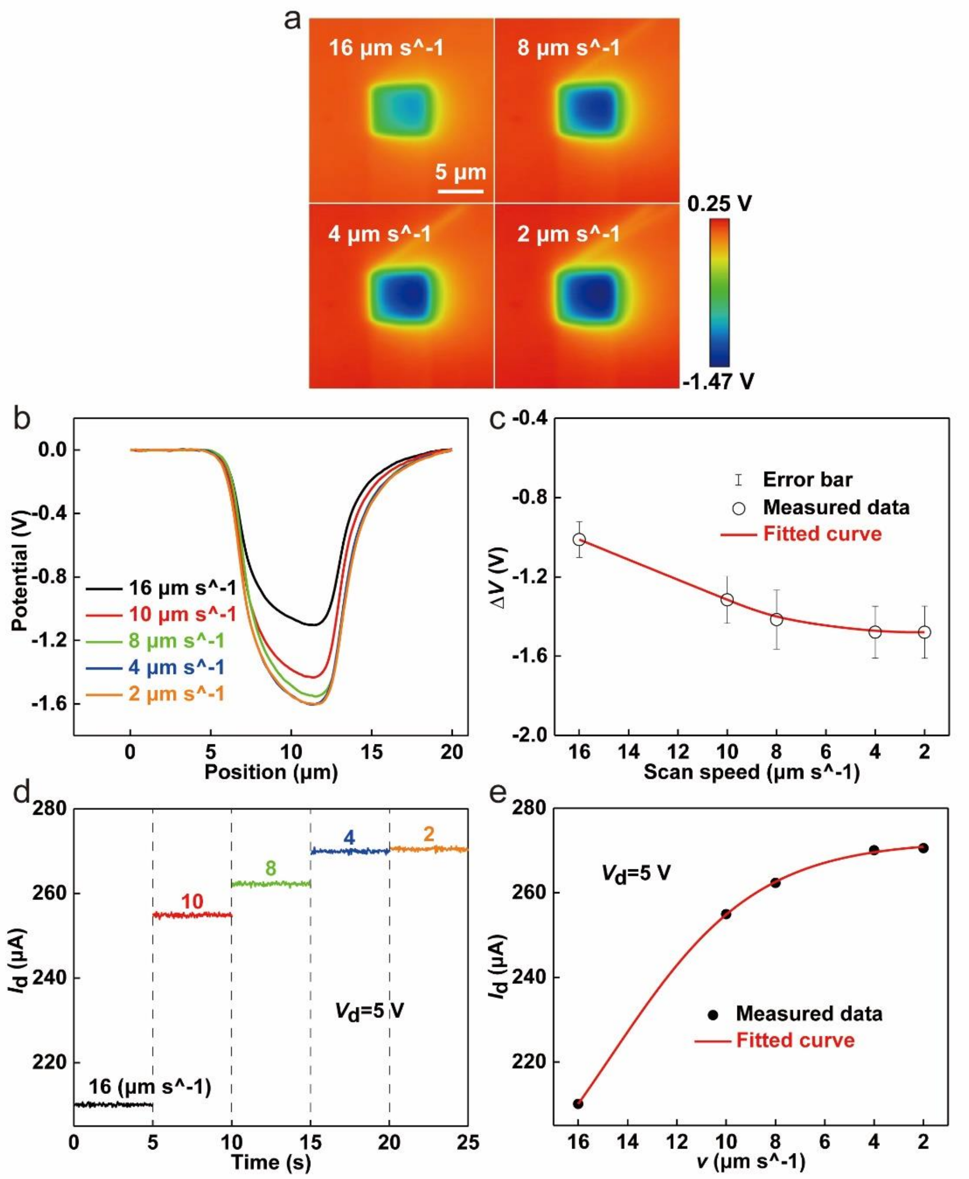

Supplementary Figure 5. The effect of scan speed on the characteristics of the NTT. a) Surface potential distributions of the NTT after regionally rubbed by the AFM tip with increasing scan speed. b) Corresponding potential distributions in cross-sectional view with different scan speeds. c) The potential difference between the rubbed and surrounding area with different scan speeds. d) I_d output characterisics at a drain voltage of 5 V with different scan speeds from 16 to 2 μm s^-1. e) The I_d-v transfer characteristics. All error bars in the figure represent s.d. of the data.

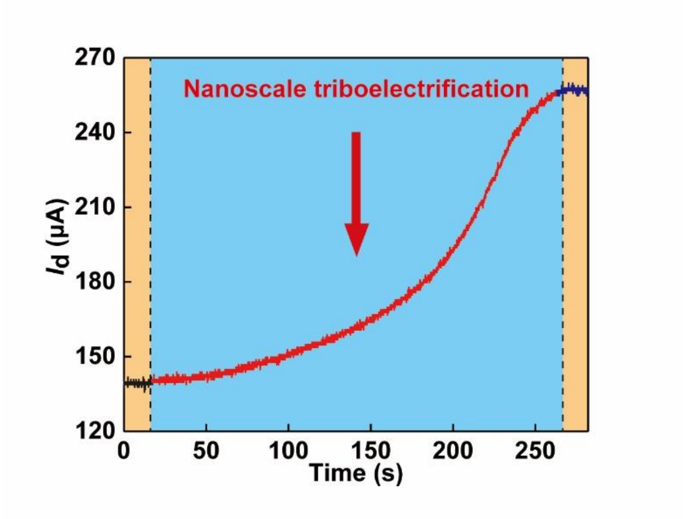

Supplementary Figure 6. Synchronous electrical monitoring of the NTT.

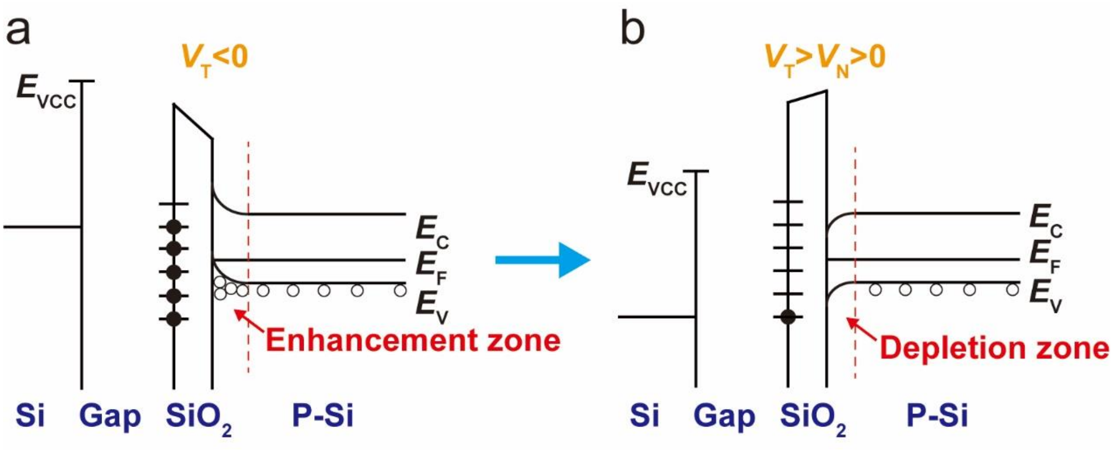

Supplementary Figure 7. Energy band diagrams of the NTT with applied tip voltage. a) The energy band diagram of the NTT when V_T<0. b) The energy band diagram of the NTT when V_T> V_N>0.

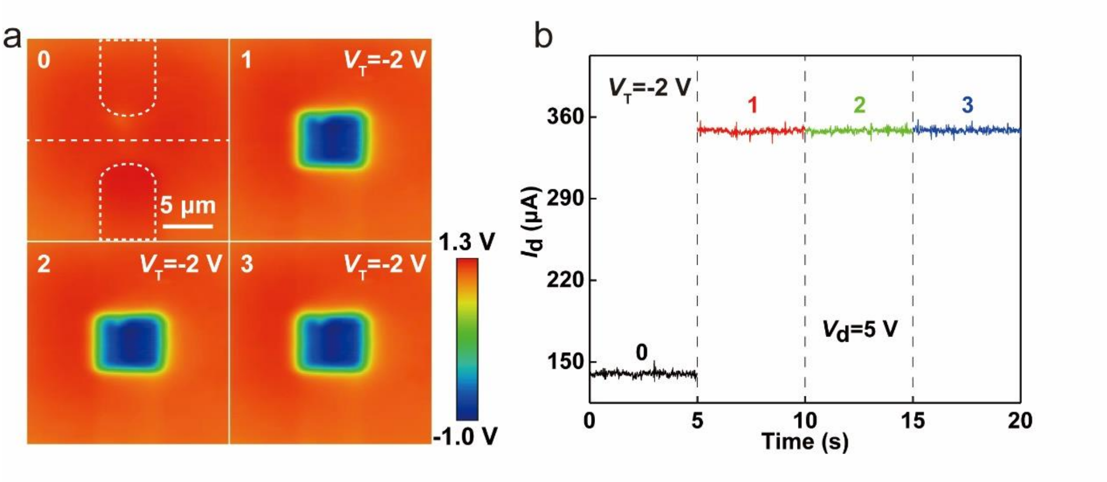

Supplementary Figure 8. The effect of contact cycles on the NTT with applied tip voltage. a) Surface potential distribution of the NTT after regionally rubbed by the AFM tip with increasing contact cycles at a tip voltage of -2 V. b) The corresponding I_d output characterisics of the NTT.

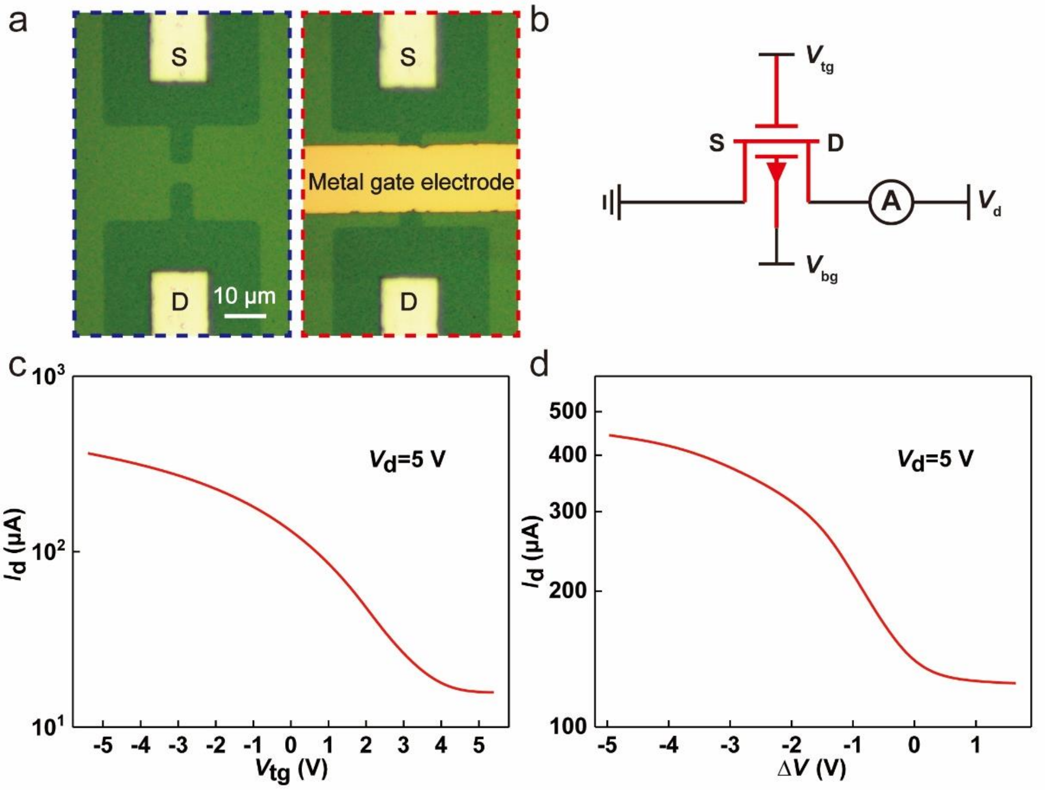

Supplementary Figure 9. Comparision between the top metal gate transistor and the NTT. a) Top-view microscope images of the transistors without top metal gate electrode and with top metal gate electrode. b) The measurement circuit for the top metal gate transistor. c) The corresponding I_d-V_{tg} transfer characteristics of the top

metal gate transistor at a drain voltage of 5 V. d) The I_d-ΔV transfer curve of the NTT at a drain voltage of 5 V.

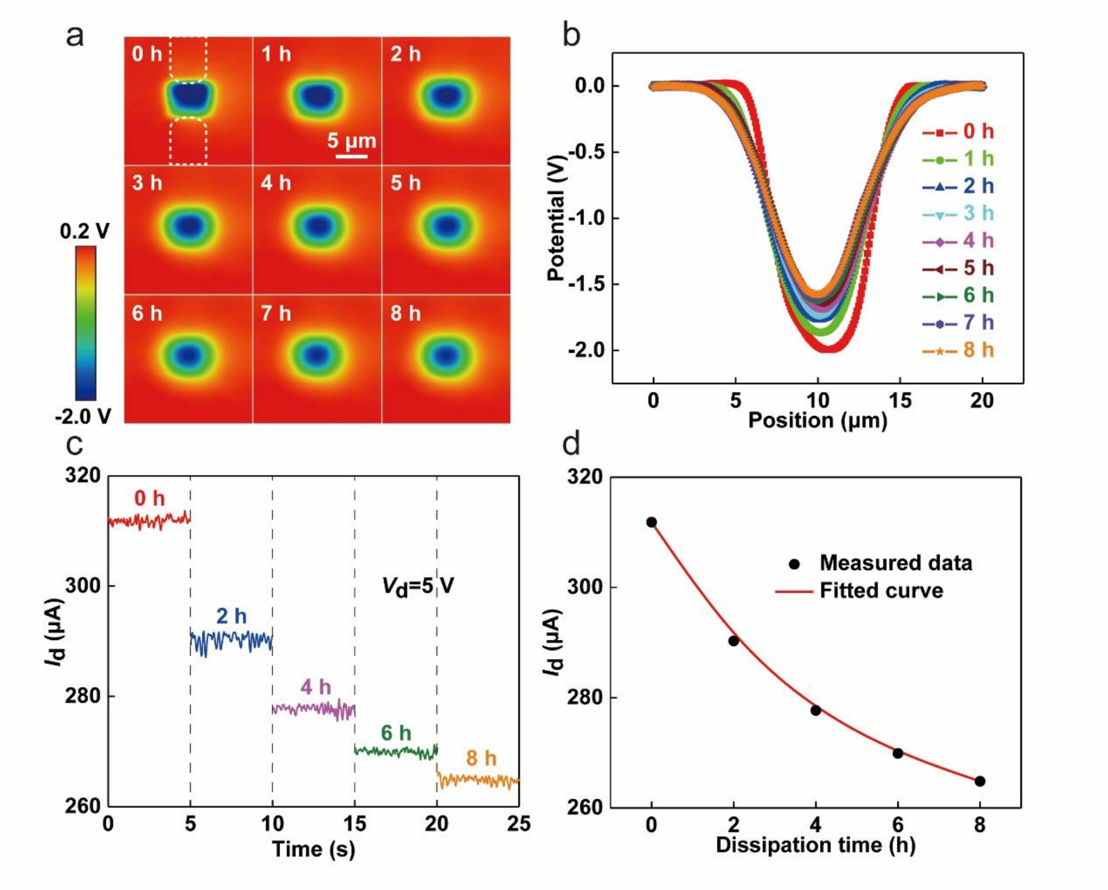

Supplementary Figure 10. The effect of charge diffusion on the NTT with applied tip voltage. a) Surface potential distributions of the NTT after regionally rubbed by the AFM tip with increasing dissipation time at a tip voltage of -2 V. b) Corresponding potential distributions in cross-sectional view. c) The corresponding I_d output characterisics of the NTT. d) The I_d-t transfer characteristics.

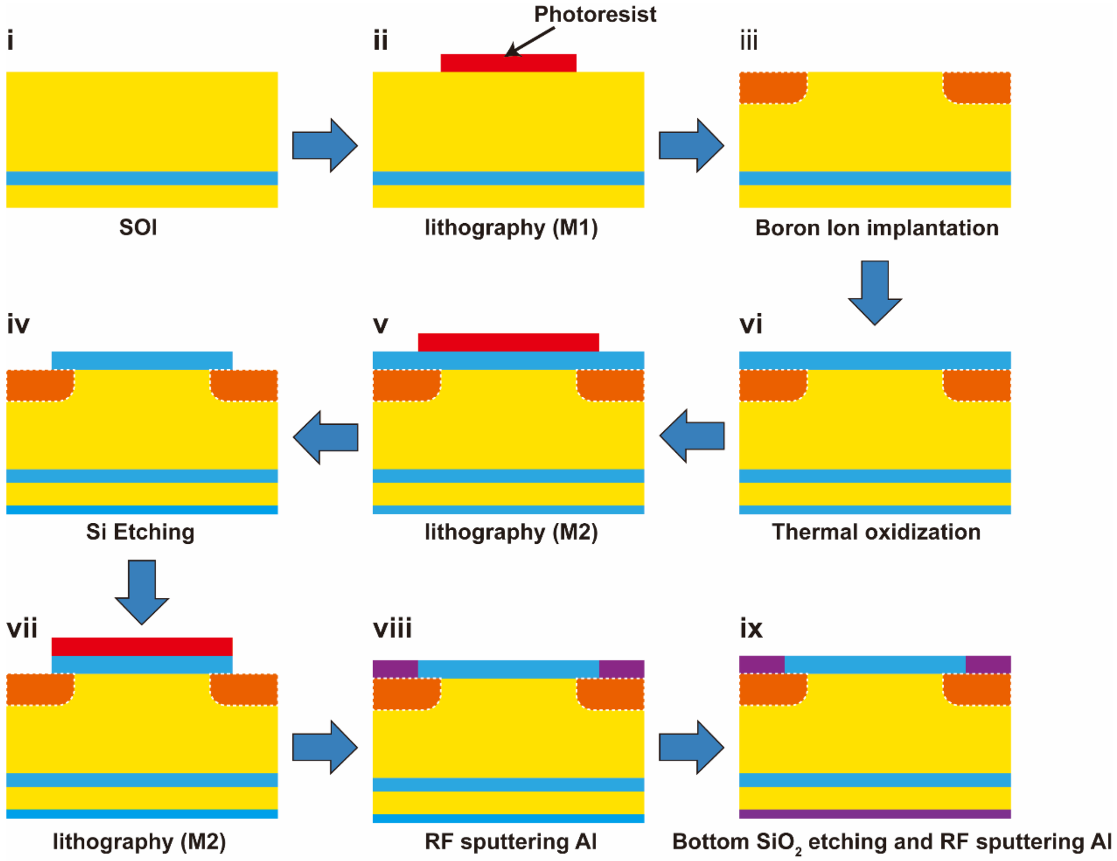

Supplementary Figure 11. Fabrication process of the NTT.

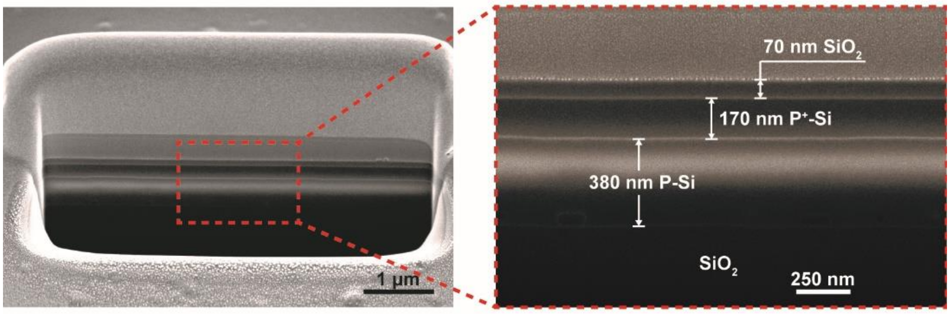

Supplementary Figure 12. Cross-sectional view of the NTT.
